# Modelling physiology of haemodynamic adaptation in short-term microgravity exposure and orthostatic stress on Earth

**DOI:** 10.1038/s41598-021-84197-7

**Published:** 2021-02-25

**Authors:** Parvin Mohammadyari, Giacomo Gadda, Angelo Taibi

**Affiliations:** 1 Department of Physics and Earth Sciences, University of Ferrara, 44122 Ferrara, Italy; 2 National Institute for Nuclear Physics (INFN), Section of Ferrara, 44122 Ferrara, Italy

**Keywords:** Circulation, Biophysics, Physiology

## Abstract

Cardiovascular haemodynamics alters during posture changes and exposure to microgravity. Vascular auto-remodelling observed in subjects living in space environment causes them orthostatic intolerance when they return on Earth. In this study we modelled the human haemodynamics with focus on head and neck exposed to different hydrostatic pressures in supine, upright (head-up tilt), head-down tilt position, and microgravity environment by using a well-developed 1D-0D haemodynamic model. The model consists of two parts that simulates the arterial (1D) and brain-venous (0D) vascular tree. The cardiovascular system is built as a network of hydraulic resistances and capacitances to properly model physiological parameters like total peripheral resistance, and to calculate vascular pressure and the related flow rate at any branch of the tree. The model calculated 30.0 mmHg (30%), 7.1 mmHg (78%), 1.7 mmHg (38%) reduction in mean blood pressure, intracranial pressure and central venous pressure after posture change from supine to upright, respectively. The modelled brain drainage outflow percentage from internal jugular veins is 67% and 26% for supine and upright posture, while for head-down tilt and microgravity is 65% and 72%, respectively. The model confirmed the role of peripheral veins in regional blood redistribution during posture change from supine to upright and microgravity environment as hypothesized in literature. The model is able to reproduce the known haemodynamic effects of hydraulic pressure change and weightlessness. It also provides a virtual laboratory to examine the consequence of a wide range of orthostatic stresses on human haemodynamics.

## Introduction

Gravity fundamentally affects the blood circulation by altering the vessels pressure, blood flow rate and volume^1–3^. Physiological effects of microgravity (μg) on cardiovascular function have been known since the first data from Soviet and American spaceflights were studied^4,5^. It is reported that about 50% of astronauts suffered from orthostatic pressure intolerance in the upright posture after space mission^5,6^. The physiological effects of hydrostatic pressure change on different parameters of cardiovascular system have largely been studied on humans and animals^3,7,8^. Indeed, there is literature about the hydrostatic stress suffered by subjects living on Earth during posture change from head-down tilt (HDT) to head-up tilt (HUT) angles up to 90°, and by exposing the subjects to weightlessness condition^7,9–15^. However, the mechanisms of cardiovascular adaptation to microgravity and posture changes are poorly understood^2,16–18^.

In response to the question “When there is no gravity pulling back the blood to heart, how the human body changes the venous drain strategy?”, there is one hypothesis that microgravity venous congestion causes a syndrome in which fluids shift away from legs towards upper limbs and head. This headward fluid shift leads to an augmentation in venous volume and cerebrospinal fluid (CSF) which brings facial puffiness and bird legs^4,7^. Other changes include increase in cardiac output^5,18^, cerebral blood inflow (CBF)^19,20^, cerebral blood flow velocity (up to 30%)^1^, cross-section area (CSA) of internal jugular vein (IJV)^2,13^, decrease in ICP and central venous pressure (CVP)^2,21^ during parabolic flight (but no change in mean arterial blood pressure (BP)^2,5,18,22^) with respect to the supine position on Earth. Noteworthy, changes in CBF and IJV-CSA are introduced as the primary signs of microgravity exposure, and changes in ICP and CVP as the secondary signs^13^.

Weightlessness environment eliminates the compressive forces that arose from tissues surrounding the vessel walls^16^. This fact contributes to increase vascular capacity and to reduce ICP and CVP to lower values than in supine position on Earth^3,4,16,18,21^. In the presence of a gravity field, the compliance properties of peripheral vessels allow them to hold a given volume of blood. This tendency remains active also in a microgravity environment, while vascular functionality and peripheral vascular resistance decrease^4,11^. Therefore, during the landing day the lower total peripheral resistance (TPR) brings intracranial hypotension and syncope in astronauts due to rapid secondary shift of blood to the lower limbs^11,23^.

Earth-based models such as HDT and water immersion are widely used to simulate the effects of microgravity on Earth and perform experiments on cardiovascular function and adaptation^3,8,18^. Another possibility is to use parabolic flights in which 20–30 s of weightlessness allow to perform measurements in such a short time^1,2,24^. However, the results highly depends on the study conditions, accuracy of the measurements and the duration of microgravity exposure^18,23^. Therefore, results from Earth-based simulations might not be used to infer conclusions about the microgravity environment^25^. Moreover, the number of clinical studies is limited due to the lack of standard data collection protocol on older missions, the limited number of space missions and studies on humans^18,26^. These restrictions, in addition to limitations on Earth-based studies, lead to a poor understanding of the cardiovascular responses to microgravity. For all these reasons, the necessity to have a reliable computed simulation tool is highlighted in this framework^16,27^.

Cardiovascular mathematical modelling is a useful method to study the human physiology and anatomy, and it is also used to plan and execute interventional procedures^16,28^. An important advantage of computational modelling is that it provides a virtual laboratory and allows the exploration of a wide range of orthostatic stresses and their complex physiological chain of events on the intra- and extracranial compartments at a low medical and computational cost^19,27,29,30^. Many valuable models of the cerebral circulation (like the works of Gisolf et al.^12^ and Buckey et al.^16^) focused on intracranial segments and related control mechanisms, by providing a simplified description of the main arterial inflow and extracranial venous return. Whole body models such as the one developed by Zhang et al.^31^ and Gallo et al.^26^ do not include brain compartments, and only the main outflow routes (IJV and VV) are included in the vascular network. Peripheral vessels play an important role in the brain and head drainage^12,32^, so that the choice to neglect them in order to simplify the model is not reasonable. Hence, the need of a comprehensive model that considers both extracranial and intracranial compartments with their interactions to adapt the whole hemodynamic system to changing environmental conditions is still not satisfied. Therefore, our overall goal is to make a hemodynamic model able to simulate the main and most reported physiological parameters such as ICP, CVP, IJV-CSA. Noteworthy, the lack of knowledge, discordant literature and measured data about hemodynamic adaptation over time during a spaceflight mission limit the computational approach^26^. To deal with these restrictions, the current model is tuned with literature reporting data of hemodynamic physiology alteration in a short-time space mission or during a parabolic flight, whose experimental conditions and measurement data are compatible.

The presented model is an advanced version of previously published simulation tools^33,34^ that were calibrated by using experimental data limited to supine and upright positions^35^. In this work, new ideas were followed to fulfil the purposes listed below:

- to simulate a full range of posture changes from HDT to supine and HUT, other than microgravity;
- to compare the simulation results with upright measured data and literature;
- to investigate the role of collateral veins in carrying out blood in case of IJV collapse (HUT) or expansion (HDT and microgravity);
- to calculate ICP and CVP changes for each simulated posture;
- to calculate flow rate changes after each posture or environmental condition change;
- to introduce new indexes useful for future investigations.

## Methods

**Figure 1:**
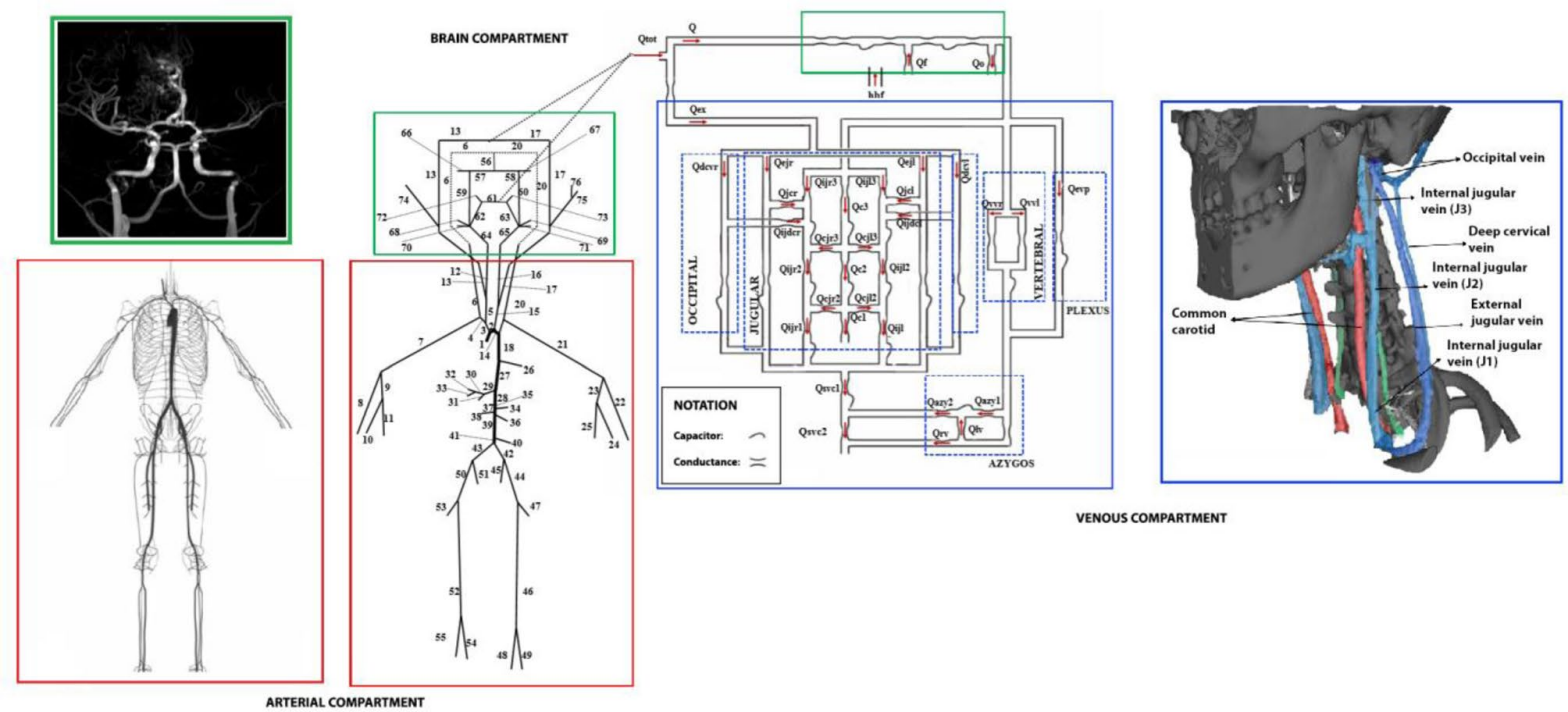
Mathematical model for the simulation of human circulation. Red boxes are the anatomy (left) and scheme (right) of the arterial tree. Green boxes are the anatomy (left), scheme (middle) of the Willis circle tree, and the scheme of the intracranial 0-D submodel (right). Blue boxes are the scheme (left) and anatomy (right) of the venous compartment.

The mathematical model consists of three compartments, linked together, that simulate the arterial, brain and venous vasculature^33^. The arterial tree is simulated through a 1-D network, while the brain and venous compartments are simulated through 0-D networks. The scheme of the model is reported in Fig. 1. We use pressure carried by anterior communicating artery in the model of the Willis circle as input to the 0-D intracranial model, and sum of pressures carried by external carotid arteries as input to the extracerebral duct. The venous compartment (blue boxes) includes veins from venous sinus to lumbo-azygos system and superior vena cava. The current version of our model is an open-loop system with a more sophisticated description of the collateral pathways, including not only the internal jugular and vertebral veins but also the external jugular veins, the vertebral plexus (including internal epidural venous plexus and deep cervical veins) and other anastomoses that carry blood to the downstream sections^33^. Therefore, the current model allows simulation of blood flows and pressures in the main vessels and collateral routes taking into account the collapsibility of the veins. In the following, the model is described with focus on the new updates to simulate the altered gravity effects on hemodynamic system. More mathematical details of the model are reported in “Supplementary Material”.

### Venous compartment adaptation

In the 0-D compartment, pressure *P* in the model is defined at every node as a function of time *t*. Equation (1) is an example of pressure variation in the venous compartment (upper segment of right IJV):

1 $$\frac{dP}{{dt}} = \frac{1}{{C_{jr3} }}\left[ {\left( {P_{vs} - P_{jr3} } \right)G_{jr3} - \left( {P_{jr3} - P_{c3} } \right)G_{cjr3} - \left( {P_{jr3} - P_{jr2} } \right)G_{jr2} } \right]$$

In this equation, *P*, *C* and *G* are the pressure, capacitance and conductance of the vessel in the upper and middle segment of the right IJV (*jr3* and *jr2*, respectively), venous sinus (*vs*), upper collateral segment (*c3*), and upper right anastomotic connection (*cjr3*). To take into account the IJV collapsibility and the dynamics of pressure change due to posture variation, *G* should vary following the CSA change. The conductance function of the segment *x* (*G*-function) has a nonlinear switch-like behaviour as in the following equation^12,36^:

2 $$\begin{aligned} & G_{x} = k_{x} \left[ {1 + \left( {\frac{2}{\pi }} \right)arctan \left( {\frac{{P_{xint} - P_{xext} }}{A}} \right) } \right]^{2} \\ & k_{x} = \frac{{V_{0}^{2} }}{{8\pi\upmu L^{3} }} \\ \end{aligned}$$

where *L* is the segment length, *V*_*0*_ is the half-maximal blood volume of the segment, *µ* is the blood viscosity, and *A* is the slope of the pressure–volume relationship or elastance^12,36,37^. The sensitivity of Eq. (2) to pressure variation due to posture changes (e.g. from supine to upright position) on a gravity field is implemented in the difference between internal *P*_*xint*_ and external *P*_*xext*_ pressure. This difference is defined as transmural pressure (*P*_*T*_ = *P*_*xint*_ − *P*_*xext*_)^34,37^. Therefore, following the *G*-function (Eq. 2), if *P*_*T*_ has positive values the IJV is fully open, while if *P*_*T*_ reaches null or negative values the IJV is partially or fully collapsed. To properly define *P*_*xext*_, we introduced the following equations:

3 $${P}_{xext}= {P}_{xhydro}+{P}_{TW}$$

4 $$P_{xhydro} = \rho \left( {\frac{g}{{g_{Earth} }}} \right)L\;sin\;\theta$$

5 $${P}_{TW}=\left[1-\left(\frac{g}{{g}_{Earth}}\right)\right]\mathrm{R}$$

where *ρ* is the blood density, *θ* is the body orientation with respect to the gravity acceleration vector of modulus *g*, *g*_*Earth*_ is the modulus of gravity acceleration vector on Earth, *P*_*xhydro*_ and *P*_*TW*_ are the hydraulic and surrounding tissue weight pressure on vessel *x*^16^. *P*_*TW*_ is related to body size and it emphasizes inter-individual differences^38^. Buckey et al.^16^ introduced the interindividual-dependent variable *R* as the radius of the body section in which external pressure is measured. We borrowed that concept to tune such parameter in accordance to the characteristics of our model. The *G*-function for supine, upright or HDT, and microgravity are defined by Eqs. from (6) to (8), respectively:

6 $${G}_{jr3}={k}_{jr3}{\left[1+\left(\frac{2}{\pi }\right)arctan\left(\frac{{P}_{vs}}{A}\right) \right]}^{2}$$

7 $${G}_{jr3}={k}_{jr3}{\left[1+\left(\frac{2}{\pi }\right)arctan \left(\frac{{P}_{vs}-{P}_{j3hydro}}{A}\right) \right]}^{2}$$

8 $${G}_{jr3}={k}_{jr3}{\left[1+\left(\frac{2}{\pi }\right)arctan\left(\frac{{P}_{vs}-{P}_{j3hydro}-{P}_{TW} }{A}\right) \right]}^{2}$$

**Figure 2:**
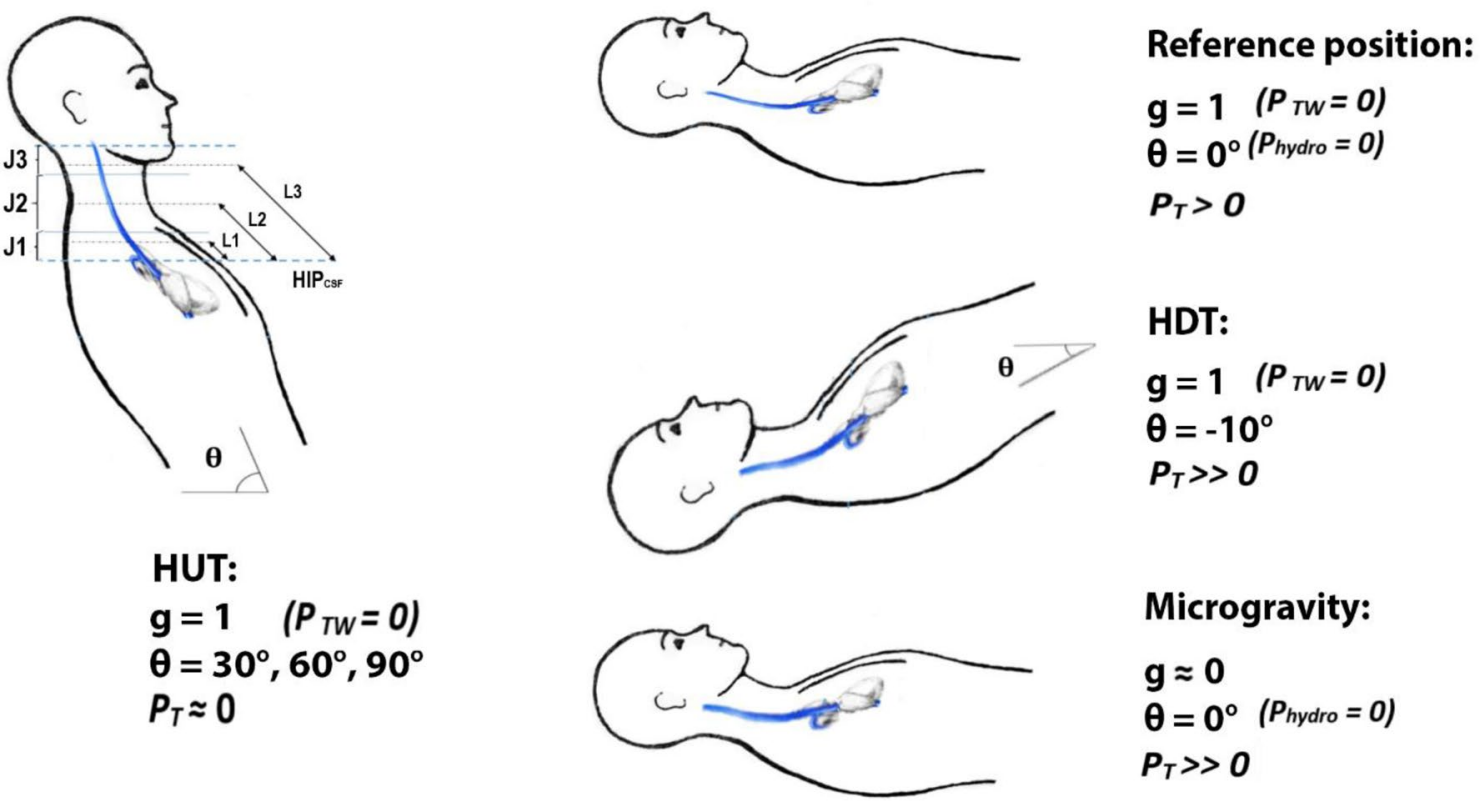
Left: Illustration of the three modelled IJV segments (J1–J3) and the corresponding vessel lengths (L1–L3) measured from the hydrostatic indifference point (HIP_CSF_) of a subject in head-up tilt position (HUT). Right: supine (reference position, top), head-down tilt (HDT, middle) and microgravity (bottom) condition. *θ* tilt angle, *g* normalized modulus of gravity acceleration vector with respect to g_Earth_, *P*_*T*_ IJV transmural pressure, *P*_*hydro*_ hydraulic pressure, *P*_*TW*_ tissue weight pressure.

In this work we assumed the same *P*_*TW*_ for all the IJV segments (J1, J2, and J3), while values of *P*_*hydro*_ were calculated from Eq. (4) by taking into account the average distance of each segment from the hydrostatic indifference point (HIP_CSF_). CSF pressure was also assumed constant. ICP gradient can be predicted according to the hydrostatic pressure gradients from the HIP_CSF_^15^. Figure 2 shows the cited IJV segments and HIP_CSF_ in a HUT subject (left). Reference position and the other conditions analyzed in this work are also reported (right).

Indeed, in case of posture variation on Earth (g = 1), the hydraulic pressure gradient causes ICP change and orthostatic stress^12,15,16^. When in microgravity (g ≈ 0), hydraulic pressure gradient has been considered null for the entire angle range^2,3,39^. Moreover, the compressive force that arose from tissues surrounding the vessel walls were eliminated in microgravity due to weightlessness. Obviously, it is not possible to properly simulate this condition on Earth.

Moreover, the model takes into account the breathing effect on the central venous pressure^40^. Breathing effect on the hydraulic properties of the IJV and VV leads to an increase in flow, even if the intracranial autoregulation mechanisms prevent alterations of cerebral perfusion due to breathing^33,41,42^. To simulate effects of the thoracic pump, the respiratory pressure pulse wave (*P*_*res*_) has been considered. The average duration time of one breathing cycle is set to 4.8 s. *P*_*res*_ was used as input to calculate the CVP pulse (*P*_*cv*_), i.e., at the end of the modelled venous pathway^43^. Taking into account the posture dependency of CVP, the *P*_*cv*_ equation were modified to include contributions of hydrostatic pressure change as well as weightlessness:

9 $$\begin{aligned} & \mathop \smallint \limits_{{P_{cv0} }}^{{P_{cv} }} dP_{cv} = \int\limits_{{t_{0} }}^{t} { \frac{1}{{C_{svc} }}\left[ {\left( {P_{svc1} - P_{cv} } \right)G_{svc1} + \left( {P_{azy} - P_{cv} } \right)G_{azy} - \left( {P_{svc2} - P_{cv} } \right)G_{svc2} } \right] dt} \\ & P_{CV0} = P_{a0} + P_{res} + P_{cv\_hydro} + P_{TW} \\ \end{aligned}$$

where *P*_*a0*_ and *P*_*cv0*_ are the arterial and central venous pressure in supine position on Earth. Since the superior vena cava (SVC) is a long vein, in our model configuration it is divided into two segments (SVC1 and SVC2). ${C}_{svc}$ is equal for both segments compliances. ${G}_{svc1} , {G}_{svc2}$ and ${G}_{azy}$ are the SVC1, SVC2 and azygous conductance, respectively. Therefore, change in *P*_*hydro*_ simulates the posture change effects from HDT to supine and upright on Earth, while *P*_*TW*_ is equal to zero. When simulating microgravity condition *P*_*hydro*_ is set to zero, while *P*_*TW*_ acts to decompress the vascular network. In this work, P_TW_ is considered to be the same for all the modelled segments of IJV.

### Brain compartment adaptation

Equations for intracranial dynamics published by Ursino and Lodi^41^ are used in the brain part of our model. These equations take into account the mass preservation at all jointing parts of the modelled vessels. At the intracranial level, the storage capacitance (*C*_*ic*_) is defined by means of the following differential equation:

10 $${C}_{ic}\frac{{dP}_{ic}}{dt} = \frac{{dV}_{pa}}{dt}+\frac{{dV}_{vi}}{dt} +{q}_{f}- {q}_{o}$$

where *P*_*ic*_ is the ICP, *V*_*pa*_ and *V*_*vi*_ are blood volume at the pial arteries and intracranial veins, *q*_*f*_ and *q*_*o*_ are the rate of formation and outflow of CSF. With the assumption of a monoexponential pressure–volume relationship for the craniospinal space, intracranial storage capacitance is inversely proportional to ICP:

11 $${C}_{ic}=\frac{1}{{k}_{E} {P}_{ic}}$$

where *k*_*E*_ is the intracranial elastance. Moreover, intracranial venous capacitance *C*_*vi*_ is defined as follow:

12 $${C}_{vi}=\frac{1}{{{k}_{ven} (P}_{v}- {P}_{ic}- {P}_{v1})}$$

where *k*_*ven*_ is a constant value, *P*_*v*_ is the cerebral venous pressure and *P*_*v1*_ is the transmural pressure. The hydraulic pial artery resistance (*R*_*pa*_) has control on the cerebrovascular mechanisms, to keep balance between pial artery volume and resistance:

13 $${R}_{pa}=\frac{{k}_{R} {C}_{pan}^{2}}{{V}_{pa}^{2}}$$

where *k*_*R*_ is a constant, and *C*_*pan*_ is a parameter to make hydraulic resistance independent from blood volume. By that, the resistance depends to the capacitance and the *k*_*R*_ value. According to the literature^11,16^, the effect of gravity on cerebral blood circulation is considered by multiplying the supine value (*k*_*x0*_) in the *k*_*E*_, *k*_*ven*_, and *k*_*R*_ parameters of Eqs. (11) to (13) to a factor as written below:

14 $${k}_{x}= {k}_{x0}(1- \alpha sin\theta +\beta {P}_{TW})$$

where *α* and *β* are parameters properly set to adjust the output, and *k*_*x*_ is one of the aforementioned parameters (*x* = {*E*, *ven*, *R*}).

### Arterial compartments adaptation

The nonlinear 1-D equations (see “Supplementary Material”) were solved by using the finite element methods^44^. Since enough information to adjust the 1-D compartments has not been reported in literature, we decided to adjust the arterial pressure *P*_*a*_ (Eq. 15) and external carotid arteries pressure *P*_*ex*_ (Eq. 16) by using *P*_*hydro*_ and *P*_*TW*_:

15 $${P}_{a} ={P}_{a0} + {P}_{xhydro}$$

16 $${P}_{ex}={P}_{ex0}+ {P}_{xhydro}+ {P}_{TW}$$

To do this, we followed the format of equation for ICP reported in the work of Magnaes^45^ (Eq. 17):

17 $${P}_{ic}={P}_{ic0}+ \rho \mathrm{g} L sin\theta$$

where *P*_*ic0*_, *P*_*a0*_, and *P*_*ex0*_ are respectively the intracranial, arterial, and external carotid pressure in supine position on Earth.

Since there are no reports of changes in systolic and mean arterial pressure in short-term spaceflight and parabolic flights studies^2,5,18^, P_TW_ is not added to Eq. (15). Moreover, plasma volume reduction (about 10%) is not considered in the study because no decrease in ICP and cerebral artery compliance was observed after short-duration spaceflight^46^. In accordance to the literature, the impedance of distal cerebral arteries would be reduced in response to mild decreases in plasma volume^46^. Hence, we decided to insert P_TW_ in the P_ex_ described in Eq. (16).

### Flow analysis

In order to analyze the flow in the head and neck network of the model, the following definitions of flow rate proposed by Zamboni et al.^32^ were used and adjusted with respect to the latest anatomical updates of the model published by Mohammadyari et al.^33^ (Fig. 1). In the following it is reported how the flow rate calculated by the arterial 1-D compartment is being used as input for the 0-D venous compartment. The head blood inflow (*Q*_*HBin*_) indicates the amount of blood entering the head through the vertebral artery (VA) and the two branches of common carotid (CC), that is the internal common carotid (IC) and external common carotid (EC):

18 $${Q}_{HBin}=\frac{{Q}_{CC}+({Q}_{IC}+{Q}_{EC})}{2}+{Q}_{VA}$$

Cerebral blood flow (*Q*_*CBF*_) indicates the incoming flow into the Willis circle of brain compartment which is supplied by IC and VA:

19 $${Q}_{CBF}={Q}_{IC}+{Q}_{VA}$$

The ECAs enter the facial and extracranial compartment and then are mainly drained by the temporal and facial veins to join external jugular vein (EJV) and the rest of neck venous network. The previous version of the venous network model^32,34^ assumed that the ECA flow rate supplies the anastomosis network of the neck. Our latest published update^33^ allows us to change the previous assumption, by stating that all the extracranial inflow (*Q*_*ex*_) is supplying the deep cervical vein (DCV) and EJV (see the venous compartment scheme in the left blue box of Fig. 1). Cerebral venous outflow (*Q*_*CVO*_) is the flow that originates from the intracranial compartment and is defined by the sum of IJVs-J3, vertebral veins (VVs) and the epidural veins (EDVs), including DCV and internal venous plexus vein (IVP)):

20 $${Q}_{CVO}={Q}_{J3}+{Q}_{VV}+{Q}_{EDV}$$

It is important to add the IVP because of the well-known role of this vein and VV as a main outstream pathway of blood from brain to SVC in the upright position, when the IJV is collapsed^10,12,32,47^. The head blood outflow (*Q*_*HBout*_) is equivalent to the sum of the flow of the IJV-J1, EJV, VV and EDV:

21 $${Q}_{HBout}={Q}_{J1}+{Q}_{VV}+{Q}_{EJV}+{Q}_{EDV}$$

In order to analyze the role of collateral vessels in head and neck drainage, the collateral-distal (*Q*_*C-D*_) and collateral-proximal flows (*Q*_*C-P*_) were defined as the outflows which directly go into the brain and neck collaterals, respectively:

22 $${Q}_{C-D}={Q}_{CBF}-{Q}_{CVO}$$

23 $${Q}_{C-P}={Q}_{HBin}-{Q}_{HBout}$$

Collateral flow index (CFI) and delta cerebral venous outflow (DCVO) are the two factors that Zamboni et al.^32^ defined to examine the percentage of blood entering the head and the normalized outflow difference:

24 $$CFI=\frac{{Q}_{C-P}}{{Q}_{HBin}}\times 100$$

25 $$DCVO_{upright} = \left[ {\left. {\left( {\frac{{Q_{HBout} }}{{Q_{HBin} }}} \right)} \right|_{supine} - \left. {\left( {\frac{{Q_{HBout} }}{{Q_{HBin} }}} \right)} \right|_{upright} } \right] \times 100$$

We also defined the DCVO_HDT_ and DCVO_μg_ to investigate the outflow differences during HDT and μg with respect to supine position, and the peripheral veins outflow index (PVI), which is equal to the percentage difference of blood that passes from the peripheral veins (except the IJV):

26 $$DCVO_{HDT} = \left[ {\left. {\left( {\frac{{Q_{HBout} }}{{Q_{HBin} }}} \right)} \right|_{supine} - \left. {\left( {\frac{{Q_{HBout} }}{{Q_{HBin} }}} \right)} \right|_{HDT} } \right] \times 100$$

27 $$DCVO_{\mu g} = \left[ {\left( {\frac{{Q_{HBout} }}{{Q_{HBin} }}} \right)|_{supine} - \left( {\frac{{Q_{HBout} }}{{Q_{HBin} }}} \right)|_{\mu g} } \right] \times 100$$

28 $$PVI=\frac{{Q}_{HBout}- {Q}_{J3}}{{Q}_{HBout}}\times 100$$

With these equations we can study the posture change effects on the haemodynamic model from − 10° HDT to 90° HUT (upright). The model parameters have been taken from previously published articles by Gadda et al.^34,35^. Model calculations are performed by the software package MATLAB-Simulink 2019b^48^.

## Results and discussion

In this work we improved the mathematical haemodynamic model to simulate the hydrostatic pressure changes and weightlessness condition. The model is calibrated in accordance to the data taken from literature. The focus was on the head and neck part of the haemodynamic system. Table 1 presents the considered literature pressure values (mean ± standard deviation) and the simulated mean values of pressure at the level of aorta (*P*_*a*_), braincase (ICP), and SVC (CVP). Noteworthy, since our model is not a closed-loop time dependent model, only the steady state conditions are simulated and presented in the following.

**Table 1:** Simulated mean pressure and literature pressure values (mean ± standard deviation) considered in this study.

	Supine	θ = 30°	θ = 60°	Upright	HDT	μg
**Simulated values**						
*P*_*a*_	100.8 ± 12.5	85.8 ± 12.5	74.8 ± 12.5	70.8 ± 12.5	106.0 ± 24.5	100.8 ± 12.5
ICP	9.5 ± 0.4	6.7 ± 0.8	3.7 ± 0.4	2.1 ± 0.4	10.5 ± 0.1	8.3 ± 0.3
CVP	4.2 ± 1.2	3.3 ± 1.3	2.7 ± 1.3	2.5 ± 1.3	4.4 ± 1.2	3.9 ± 1.1
*P*_*j3*_	4.8 ± 0.9	4.4 ± 0.8	4.5 ± 0.8	4.6 ± 0.8	5.0 ± 0.9	4.7 ± 0.9
*P*_*j2*_	4.6 ± 0.9	4.0 ± 0.9	3.9 ± 0.6	3.8 ± 0.6	4.9 ± 0.9	4.5 ± 0.9
*P*_*j1*_	4.4 ± 1.1	3.5 ± 1.2	2.8 ± 1.3	2.6 ± 1.3	4.6 ± 1.0	4.2 ± 1.0
**Lindén et al.**^24^						
ICP	10.5 ± 1.5	N/A	N/A	− 0.8 ± 3.8	− 9°: 15.8 ± 1.2	N/A
**Lawley et al.**^2^						
*P*_*a*_	~ 100	N/A	N/A	Δ = − 13.0 ± 7.1	N/A	Δ = − 1.9 ± 5.1
ICP	15 ± 2	N/A	N/A	4 ± 1	− 6°: 15 ± 4 (Δ = 1.9 ± 0.5)	13 ± 2
CVP	7 ± 3	N/A	N/A	2 ± 3	− 6°: unchanged (Δ = − 0.5 ± 0.5 )	4 ± 2
**Eklund et al.**^49^						
ICP	10.5 ± 1.5	N/A	N/A	− 0.8 ± 3.8	− 9°: 15.8 ± 1.3	N/A
**Petersen et al.**^50^						
*P*_*a*_	103 ± 19	10°: 88 ± 9	20°: 87 ± 7	87 ± 8	− 10°: 86 ± 11	N/A
ICP	9.4 ± 3.8	4.8 ± 3.6	1.3 ± 3.6	− 2.4 ± 4.2	14.3 ± 4.7	N/A
**Qvarlander et al.**^15^						
ICP	11.0 ± 2.1	27°: 2.3 ± 2.5	57°: − 1.0 ± 3.0	71°: − 1.8 ± 3.2	N/A	N/A
**Mekis and Kamenik**^51^						
*P*_*a*_	72.9 ± 11.6	20°: 65.1 ± 13.1	N/A	N/A	− 20°: 85.4 ± 14.0	N/A
CVP	8.1 ± 4.9	20°: 4.5 ± 3.8	N/A	N/A	− 20°: 12.7 ± 4.7	N/A
**Buckey et al.**^16^** (mathematical model)**						
ICP	6.1	N/A	N/A	N/A	N/A	2.4
CVP	3.5	N/A	N/A	N/A	N/A	− 6

All values are reported in mmHg. We refer to pressure gradient ∆ when absolute values are not reported by cited authors.

*θ* tilt angle, *HDT* head-down tilt, *μg* microgravity, *P*_*a*_ arterial pressure, *ICP* intracranial pressure, *CVP* central venous pressure.

We see from Table 1 that subject posture affects the measured pressure values. In particular, transition from supine to upright causes a pressure decrease, while transition from supine to HDT causes a pressure increase. Arterial BP is hypothesised to be about 100 mmHg in supine, to decrease by increasing the HUT angle till the value of 70 mmHg in upright, and to increase to 105 mmHg at a HDT angle of 6°^7^. Also, it is reported that in microgravity it remains unaffected with respect to measurements performed in the supine position before the flight^5,22^ around the mean value of 100 mmHg^39^. Petersen et al.^50^ reported a 17 mmHg drop in arterial BP when passing from supine to − 10° HDT (Table 1). Our model shows good agreement with aforementioned literature when simulations of arterial BP in HUT, − 10° HDT, and weightlessness condition are performed. As reported in Table 1, the mean simulated arterial BP decreases of 30 mmHg in upright with respect to the supine position, while it increases of 5 mmHg in HDT, and it remains constant in microgravity condition.

ICP values are frequently reported in literature, and a significant reduction during HUT posture change from supine is always measured^2,15,24,49,50^. Moreover, the normal range of ICP for a supine adult is about 7–15 mmHg, and in general less than 20 mmHg^52^. Literature in Table 1 reported ICP change in sitting/upright in comparison with supine in the range of 12.8 mmHg (116%) to 11 mmHg (73%) (Qvarlander et al.^15^ and Lawley et al.^2^). Our simulated ICP decreases of about 7.4 mmHg (78%) in upright with respect to the supine position, which is within the aforementioned literature range, while it increases of 1 mmHg in HDT. Lawley et al.^2^ reported a slight increase (i.e. Δ = 1.8 ± 0.5 mmHg) for − 6° HDT which is comparable with our finding. However, Lindén et al.^24^ and Eklund et al.^49^ measured the same increase in the mean value of ICP (5.3 mmHg) for − 9° HDT. Such high pressure difference cannot be calculated by the known hydrostatic pressure equation implemented in the model (Eq. 4). The difference with data reported in literature might be due to the measurement dependency to experimental conditions^16,23,38^. Table 1 also shows that ICP in microgravity environment has a slight reduction (Δ = − 1.9 ± 5.1) with respect to supine position on Earth^2^. During simulation of microgravity condition, our model calculates a ICP of 8.3 mmHg, and a ICP reduction of 1.2 mmHg with respect to supine position on Earth, in very good agreement with measurement data during 5–10 parabolic flights reported by Lawley et al^2^. Besides, measurements of CVP reported by Lawley et al.^2^ indicated that such value does not change when passing from supine to − 6° HDT. Mekis and Kamenik^51^ measured a 4.6 mmHg augmentation for − 20° HDT. Results from our model shows a 1.6 mmHg reduction of CVP in upright with respect to the supine position on Earth, while it is almost unchanged in − 10° HDT. Noteworthy, the equation used in the model to calculate CVP^33–35^ is only influenced by *P*_*hydro*_ and *P*_*TW*_ on the initial value (*P*_*cv0*_), then CVP is affected mainly by IJV changes as explained in the “Methods” (Eqs. 1–8) and “Supplementary Material Section”.

Gallo et al. reported 5.1% reduction for simulated CVP, in accordance with observed early spaceflights data^21,26^. Simulated CVP in microgravity condition shows a slight decrease (7.6%) with respect to the simulation in supine position on Earth. Moreover, their CVP value in microgravity is lower than in upright and supine position. Trend of our model results in different conditions is in very good agreement with this and other literature data^2,18,21,26,33–35,51^. Moreover, simulated CVP in HDT is higher than in microgravity^53,54^; this result suggests that the upright fluid shift does not contribute to CVP reduction in microgravity, while the gravitational unloading of the *P*_*TW*_ plays a key role. The model also allows to compare the pressure variation in the three segments of left IJV for HUT, HDT and μg with respect to the supine condition, considering that J3 is the farthest segment from HIP_CSF_, and that pressure in each segment of IJV and in each condition is calculated accordingly (i.e. *P*_*J3hydro*_ > *P*_*J2hydro*_ > *P*_*J1hydro*_). Moreover, due to the relatively larger CSA of segment J1, this section exhibits the lowest pressure value in supine, and consequently the higher pressure variation in upright and HDT (ΔP_J1_ > ΔP_J2_ > ΔP_J3_). The IJV pressure value decreases when passing from supine to upright and increase at − 6° HDT. In microgravity it is higher than in upright, and comparable to the value in supine position. Therefore, the IJV pressure change follows the pattern of simulated CVP variation in different conditions.

**Figure 3:**
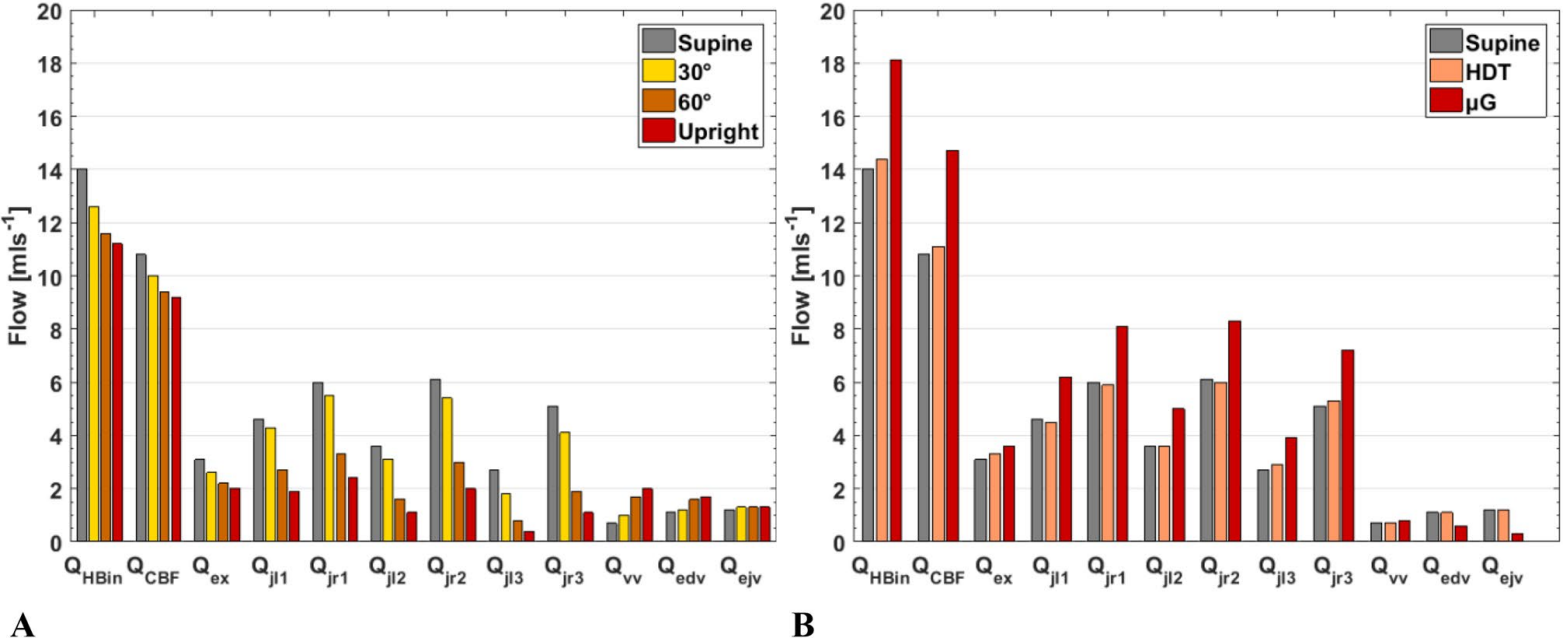
Comparison of simulated flow rates for different posture conditions on Earth (1 g, left), supine and HDT on Earth (1 g), and weightlessness (μg, right). *Q*_*HBin*_ total blood flow to the head, *Q*_*CBF*_ cerebral blood inflow, *Q*_*ex*_ extracerebral blood flow, *Q*_*jl1*_ flow in the lower segment of left IJV, *Q*_*jr1*_ flow in the lower segment of right IJV, *Q*_*jl2*_ flow in the middle segment of left IJV, *Q*_*jr2*_ flow in the middle segment of right IJV, *Q*_*jl3*_ flow in the upper segment of left IJV, *Q*_*jr3*_ flow in the upper segment of right IJV, *Q*_*vv*_ flow in the VV, *Q*_*edv*_ flow in epidural vein, *Q*_*ejv*_ flow in external jugular vein.

In Fig. 3 the simulated flow rates are reported and compared for different vessels, posture and gravity conditions. Because of the low flow rate at the collateral vessels, in this work we reported the vertebral vein flow rate as the summation of flow at right and left vertebral vein. The same strategy was used for epidural and external jugular veins. Zamboni et al.^32^ stated that the blood flow at each segment of IJV is more than that of VVs or EDV. Moreover, in the publication of Gadda et al.^34^ it is reported that blood flow in J3 is lower than in the other segments (*Q*_*J1*_ > *Q*_*J2*_ > *Q*_*J3*_) with the subject in standing position. Therefore, it means that the higher segment (with respect to the HIP_CSF_ zero level) is more collapsed. The simulated flow rates reported in Fig. 3 (summation of left and right, J1: 10.6, J2: 9.7 and J3: 7.8 ml/s) are then in good agreement with the experimental data reported by Zamboni et al.^32^ and Gadda et al.^34^ (Table 2). Furthermore, it is proven that an increase of the tilt angle contributes to the IJVs collapse and expansion of peripheral veins as a compensatory mechanism^12,14,15,32^. In this regard, the *G*-function for all the simulated neck veins has been integrated in the model (Eq. 2), so as to allow the model to simulate the different degree of collapse at IJV segments, other than the slight dilatation in VV, IVP, and DCV. In other words, the model takes into account the increase of IJV resistance and the TPR decrease during upright simulations. Figure 3A shows simulated flow rates for different posture condition on Earth. We can see that flow is properly driven to the peripherals when in upright, in good agreement with literature reports. The model simulates a reduction in *Q*_*HBin*_, *Q*_*CBF*_, *Q*_*ex*_, and IJVs flow rates, and augmentation of VVs and EDV flow rates. The extracranial veins such as EJVs do not show significant changes. Regarding the total *Q*_*HBin*_ and *Q*_*CBF*_ values, reductions after 90° posture change from supine to upright are about 20% and 15% (from 14.0 and 10.8 ml/s in supine to 11.2 and 9.2 ml/s in upright), respectively. The modelled *Q*_*CBF*_ reduction is comparable with the 12% reduction reported by Alperin et al.^9^. Similarly, Zhang and Levine^17^ measured 10–20% reduction in CBF velocity in the middle cerebral artery. Moreover, Sato et al.^19^ and Serrador and Freeman^20^ showed that the inflow increase is due to the functionality of central arteries, and not to the peripheral ones during upright tilt. This means that the change in flow rate can be directly related to flow velocity variation, and our 20% simulated reduction in Q_HBin_ is then in very good agreement with literature. In Fig. 3B the environmental variables are HDT angle and weightlessness. Considering the fluid shift in the head-down position and microgravity, the augmented *Q*_*HBin*_ leads to an outflow increase. Such increased outflow is mainly supported by the IJVs, with a consequent increase of IJV-CSA, since the flow rate can be calculated by multiplying CSA and mean velocity (see Eq. 22 in “Supplementary material”)^18,36,37^. Moreover, the 30% reduction in blood flow velocity during parabolic flight reported by Bondar et al.^1^ can be interpreted as 30% reduction in total blood inflow which is the same as our model response reduction (4 ml/s). For what concerns effects of microgravity, literature highlighted the role of tissue weight on pressure changes and flow rates^3,7,16^. HDT is only able to model the headward fluid shift in inherently ground-based simulation condition. Besides, astronauts after a long-term space mission show puffy faces, and thus suggesting that the fluid volume in head and neck is augmented also by the microgravity environment rather than exclusively by HDT. Although we are modelling the short-term microgravity exposure, the unloading tissue weight effect implies difference between Earth-based HDT simulation and real microgravity^16,53,54^. The model simulates higher *Q*_*HBin*_ (+ 4.1 ml/s), Q_CBF_ (+ 3.9 ml/s), and IJV (+ 1.7 ml/s) flow rate in microgravity with respect to supine position on Earth. Therefore, model results are satisfactory and in good agreement with what highlighted by literature reports and predictions^5,55^. Niggemann et al.^14^, Valdueza et al.^56^ and Ciuti et al.^10^ emphasized the important role of peripheral veins such as EDV in venous drainage in the upright position. However, due to the difficulty to properly assess flow rate in small and inaccessible veins, such value has not been quantitively reported^14^. Therefore, the calculated *Q*_*EDV*_ can be assumed as a predicted mean value.

**Table 2:** Simulated mean flows and literature flow values (mean ± standard deviation) considered in this study.

	Primary pathway (IJV)	Secondary pathway	
**Simulated values**^**a**^			
Supine	9.4 ± 1.8	VV: 0.7 ± 0.3	EDV: 1.1 ± 0.6
30°	8.1 ± 1.2	VV: 1.0 ± 0.2	EDV:1.2 ± 0.3
60°	4.4 ± 0.4	VV: 1.7 ± 0.2	EDV:1.6 ± 0.3
Upright	3.0 ± 0.3	VV: 2.0 ± 0.2	EDV:1.7 ± 0.4
HDT (− 10°)	9.4 ± 1.9	VV: 0.7 ± 0.3	EDV: 1.1 ± 0.7
Microgravity	12.9 ± 1.9	VV: 0.8 ± 0.4	EDV: 0.6 ± 0.4
**Ciuti et al.**^10^			
Supine	5.6 ± 3.9	VV: 0.6 ± 2.8	
Upright	2.0 ± 2.1	VV: 0.4 ± 0.4	
**Zamboni et al.**^32^^**a**^			
Supine	6.2 ± 4.5	VV: 0.6 ± 0.5	
Upright	5.0 ± 5.3	VV: 1.1 ± 0.9	
**Alperin et al.**^9^			
Supine	10.2 ± 2.4	N/A	
Upright	5.1 ± 4.4	N/A	
**Cirovic et al.**^57^			
Supine	15.5 ± 8.0	N/A	
Upright	6.2 ± 3.2	N/A	
**Valdueza et al.**^56^			
Supine	11.7 ± 4.5	VV: 0.7 ± 0.3	
15°	2.5 ± 2.2	VV: 1.5 ± 1.0	
30°	2.3 ± 3.3	VV: 1.8 ± 1.2	
45°	1.8 ± 2.5	VV: 2.2 ± 1.2	
Upright	1.2 ± 1.7	VV: 3.5 ± 2.0	

All values are reported in ml/s.

*IJV* internal jugular vein, *VV* vertebral vein, *EDV* epidural vein (summation of VV and DCV), *HDT* head-down tilt.

^a^The IJV flow is the average value of the three segments (J1, J2, and J3).

Table 2 shows the mean flow rates computed by our model and measurements (mean ± standard deviation) from literature. Valdueza et al.^56^ reported a 90% reduction of IJV flow and a 425% increase of VV flow in upright posture compared to supine. Ciuti et al.^10^, Cirovic et al.^57^ and Alperin et al.^9^ measured 64%, 60% and 50% reduction in the IJV flow rate during the transition from supine to sitting posture, respectively. Zamboni et al.^32^ measured flow in the three segments of IJV. They reported a flow reduction of 20%, 37%, and 34% in J1, J2, and J3 respectively, with an overall IJV average reduction of 37%. They also measured a 26% increase of VV flow after the posture change from supine to upright. The presented model simulates a IJV flow reduction of 68% (from 9.4 ml/s supine to 3.0 ml/s in upright). Conversely, the modelled secondary pathway (sum of VVs and DCVs flows) shows an increase of 108% (from 1.8 ml/s supine to 3.7 ml/s in upright) as compensatory system response of the model. Alperin et al.^9^ reported the normalized IJV outflow with respect to the total cerebral blood flow in both positions of supine and upright as 75% ± 14% and 42% ± 34%, respectively. The same values calculated by our model are 67% and 26%. Such results are comparable, if we consider the large uncertainty in the experimental values. Model results did not show significant differences in the mean flow rate values when in supine and after HDT, however they show a significant increase of IJV flow rate in microgravity condition. On the other hand, the modelled secondary pathway flows did not change neither in HDT nor in microgravity conditions. Arbeille et al.^58^ reported an IJV volume increase during a long-term space mission. They measured a volume increase of 178% and 225% after 15 days and 4–5.5 months, respectively. Comparison with our results (increase of 37% in total IJV flow rate) shows that the presented model is able to simulate the short-term microgravity exposure condition. To our knowledge, there is no literature report about direct measurements of venous flow rates for what concern HDT and microgravity.

Finally, the simulated mean values of CFI, DCVO and PVI indexes are reported in Table 3, and compared with results from Zamboni et al.^32^ who proposed the first two indexes. The new index of PVI indicates that, in a simulation of supine position, 42% of the brain and head outflow is passing through peripheral veins. This index is highly sensitive to posture change so that in upright, HDT (− 10°), and microgravity it increases up to 78%, 57%, and 53%, respectively. Our model simulations also show that, in supine position, 3% of the blood entering into the head and neck circulation goes to the collateral (CFI). This percentage increases up to 13% in upright position, 8% in HDT, and 11% in microgravity condition. The DCVO index emphasises the importance of posture change and weightlessness on the blood circulation since it represents the normalized outflow difference with respect to the supine reference condition. Zamboni et al.^32^ reported this index just for the upright posture. Table 3 shows that our simulated values are in good agreement with the experimental findings of Zamboni et al.^32^.

**Table 3:** Simulated and literature mean indexes.

	Supine	Upright	HDT	μg
**CFI**				
Simulated values	3%	13%	8%	11%
Zamboni et al.^32^	1 ± 3%	9 ± 19%	N/A	N/A
**DCVO**				
Simulated values	N/A	10%	5%	7%
Zamboni et al.^32^	N/A	5 ± 10%	N/A	N/A
**PVI**				
Simulated values	42%	78%	57%	53%

*HDT* head-down tilt, *μg* microgravity, *CFI* collateral flow index, *DCVO* delta cerebral venous outflow, *PVI* peripheral veins outflow index.

However, there are limitations in such simulations. Since the space environment is not easily accessible, there is not a united protocol to measure the physiological parameters in astronauts and then tune the simulation model accordingly. The other limitation mentioned by literature is that measurement results are highly dependent on the study conditions, such as the duration of the spaceflight and inter-individual differences (e.g. body size and weight)^16,38,39^. Moreover, this current version of the model is not able to follow the haemodynamic changes and body adaptation concerning the long-term mission and transient condition (in-flight).

## Conclusions

In this study, we presented an updated version of a mathematical model based on physiological parameters to study human blood circulation, with focus to head and neck vasculature. Our model is the first full-body map that provides comprehensive insights into the effect of microgravity on human body physiology, including the effects of hydraulic pressure change and weightlessness. The microgravity physiology is a complex subject, hence, the aim of the present work was to show the capability of this model to be used as a helpful tool in the process of understanding the consequence of any hydrostatic change in cardiovascular physiology. Noteworthy, this is a multiscale model tuned by literature data and it is not able to assess any change in human haemodynamic system. The main capability and purpose of such modelling is the calculation of physiological changes if the pertinent equations and parameters are correctly introduced. Another advantage of the presented model is the possibility to modify the equations of the 0-D compartment in order to simulate additional orthostatic stress causes. Limitations aside, this model offers the possibility to investigate counteracting procedures to reduce the orthostatic stress in returning astronauts, and it will be the subject of future work.

## Supplementary Information

Supplementary Information.

## Abbreviations

A: Slope of the pressure–volume relationship (elastance)
BP: Blood pressure
C: Capacitance
C3: Upper collateral segment
CC: Common carotid artery
CFI: Collateral flow index
C_IC_: Intracranial capacitance
C_J3_: Upper anastomotic connection
CSA: Cross-section area
CSF: Cerebrospinal fluid
C_vi_: Intracranial venous capacitance
CVP: Central venous pressure
DCV: Deep cervical vein
DCVO: Delta cerebral venous outflow
EC: External carotid artery
EDV: Epidural vein
EJV: External jugular vein
g: Modulus of gravity acceleration vector
G: Conductance
g_Earth_: Modulus of gravity acceleration vector on Earth
HDT: Head-down tilt
HIP: Hydrostatic indifference point
HUT: Head-up tilt
IC: Internal carotid artery
ICP: Intracranial pressure
IJV: Internal jugular vein
IVP: Internal venous plexus
J1: Internal jugular vein, segment 1
J2: Internal jugular vein, segment 2
J3: Internal jugular vein, segment 3
k_E_: Intracranial elastance
L: Vessel segment length
µ: Blood viscosity
µG: Microgravity
P: Pressure
P_a_: Arterial pressure
P_a0_: Arterial supine pressure on Earth
P_ex_: External carotid artery pressure
P_ex0_: External carotid supine pressure on Earth
P_ext_: External pressure
P_hydro_: Hydraulic pressure
P_ic0_: Intracranial supine pressure on Earth
P_int_: Internal pressure
P_T_: Transmural pressure
P_TW_: Tissue weight pressure
P_v_: Cerebral venous pressure
PVI: Peripheral veins outflow index
Q: Flow rate
Q_CBF_: Cerebral blood flow
Q_C-D_: Collateral-distal flow
Q_C-P_: Collateral-proximal flow
Q_CVO_: Cerebral venous outflow
Q_ex_: Extracranial inflow
q_f_: CSF formation rate
Q_HBin_: Head blood inflow
Q_HBout_: Head blood outflow
q_o_: CSF outflow rate
R: Radius of the body section
ρ: Blood density
R_pa_: Pial artery resistance
SVC: Superior vena cava
θ: Body orientation with respect to the gravity acceleration vector
TPR: Total peripheral resistance
V_0_: Parameter related to blood volume
VA: Vertebral artery
V_vi_: Blood volume at intracranial veins
V_Pa_: Blood volume at pial arteries
VS: Venous sinus
VV: Vertebral vein

## References

[1] Bondar RL (1991). Cerebral blood flow velocities by transcranial Doppler during parabolic flight. J. Clin. Pharmacol..

[2] Lawley JS (2017). Effect of gravity and microgravity on intracranial pressure. J. Physiol..

[3] Marshall-Goebel K, Damani R, Bershad EM (2019). Brain physiological response and adaptation during spaceflight. Neurosurgery..

[4] Nicogossian AE (1993). Space Physiology and Medicine.

[5] Norsk P (2006). Vasorelaxation in space. Hypertension.

[6] Mader TH (2011). Optic disc edema, globe flattening, choroidal folds, and hyperopic shifts observed in astronauts after long-duration space flight. Ophthalmology.

[7] Hargens AR, Vico L (2016). Long-duration bed rest as an analog to microgravity. J. Appl. Physiol..

[8] Keil LC, McKeever KH, Skidmore MG, Hines J, Severs WB (1992). The effect of head-down tilt and water immersion on intracranial pressure in nonhuman primates. Aviat. Space Environ. Med..

[9] Alperin N, Lee SH, Sivaramakrishnan A, Hushek SG (2005). Quantifying the effect of posture on intracranial physiology in humans by MRI flow studies. J. Magn. Reson. Imaging..

[10] Ciuti G, Righi D, Forzoni L, Fabbri A, Pignone AM (2013). Differences between internal jugular vein and vertebral vein flow examined in real time with the use of multigate ultrasound color Doppler. AJNR Am. J. Neuroradiol..

[11] Convertino VA, Cooke WH (2007). Vascular functions in humans following cardiovascular adaptations to spaceflight. Acta Astronaut..

[12] Gisolf J (2004). Human cerebral venous outflow pathway depends on posture and central venous pressure. J. Physiol..

[13] Martin DS (2016). Internal jugular pressure increases during parabolic flight. Physiol. Rep..

[14] Niggemann P (2011). Position dependent changes of the cerebral venous drainage—Implications for the imaging of the cervical spine. Cent. Eur. Neurosurg..

[15] Qvarlander S, Sundström N, Malm J, Eklund A (2013). Postural effects on intracranial pressure: Modeling and clinical evaluation. J. Appl. Physiol..

[16] Buckey, J. C. *et al.* The importance of tissue weight and tissue compressive forces in human spaceflight. Paper presented at the international astronautical congress, Adelaide, Australia, 25–29 September 2017.

[17] Zhang R, Levine BD (2007). Autonomic ganglionic blockade does not prevent reduction in cerebral blood flow velocity during orthostasis in humans. Stroke.

[18] Norsk P (2020). Adaptation of the cardiovascular system to weightlessness: Surprises, paradoxes and implications for deep space missions. Acta Physiol. (Oxf.).

[19] Sato K (2012). Blood flow in internal carotid and vertebral arteries during orthostatic stress. Exp. Physiol..

[20] Serrador JM, Freeman R (2017). Enhanced cholinergic activity improves cerebral blood flow during orthostatic stress. Front. Neurol..

[21] Buckey JC (1996). Central venous pressure in space. J. Appl. Physiol..

[22] Verheyden B, Liu J, Beckers F, Aubert AE (2009). Adaptation of heart rate and blood pressure to short and long duration space missions. Respir. Physiol. Neurobiol..

[23] Zhang LF (2001). Vascular adaptation to microgravity: What have we learned?. J. Appl. Physiol..

[24] Lindén C (2018). Normal-tension glaucoma has normal intracranial pressure: A prospective study of intracranial pressure and intraocular pressure in different body positions. Ophthalmology.

[25] Christensen NJ, Heer M, Ivanova K, Norsk P (2005). Sympathetic nervous activity decreases during head-down bed rest but not during microgravity. J. Appl. Physiol..

[26] Gallo C, Ridolfi L, Scarsoglio S (2020). Cardiovascular deconditioning during long-term spaceflight through multiscale modeling. NPJ Microgravity.

[27] Mader G, Olufsen M, Mahdi A (2015). Modeling cerebral blood flow velocity during orthostatic stress. Ann. Biomed. Eng..

[28] Slesnick TC (2017). Role of computational modelling in planning and executing interventional procedures for congenital heart disease. Can. J. Cardiol..

[29] Diaz-Artiles A, Heldt T, Young LR (2016). Effects of artificial gravity on the cardiovascular system: Computational approach. Acta Astronaut..

[30] Sharp MK, Batzel JJ, Montani J (2013). Space physiology IV: Mathematical modeling of the cardiovascular system in space exploration. Eur. J. Appl. Physiol..

[31] Zhang X, Noda S, Himeno R, Liu H (2017). Gravitational effects on global hemodynamics in different postures: A closed-loop multiscale mathematical analysis. Acta Mech. Sinica.

[32] Zamboni P (2013). An ultrasound model to calculate the brain blood outflow through collateral vessels: A pilot study. BMC Neurol..

[33] Mohammadyari P, Gadda G, Taibi A, Munuera Del Cerro J (2020). Paediatric haemodynamic modelling: Development and experimental validation using quantitative flow MRI. Eur. Radiol. Exp..

[34] Gadda G (2015). A new hemodynamic model for the study of cerebral venous outflow. Am. J. Physiol. Heart Circ. Physiol..

[35] Gadda G (2016). Validation of a hemodynamic model for the study of the cerebral venous outflow system using MR imaging and echo-color Doppler data. AJNR Am. J. Neuroradiol..

[36] Fung YC (1997). Biomechanics. Circulation.

[37] Braakman R, Sipkema P, Westerhof N (1989). A dynamic nonlinear lumped parameter model for skeletal muscle circulation. Ann. Biomed. Eng..

[38] Otsuka K (2016). Long-term exposure to space's microgravity alters the time structure of heart rate variability of astronauts. Heliyon.

[39] Zhang LF, Hargens AR (2018). Spaceflight-induced intracranial hypertension and visual impairment: Pathophysiology and countermeasures. Physiol. Rev..

[40] Gadda G, Majka M, Zieliński P, Gambaccini M, Taibi A (2018). A multiscale model for the simulation of cerebral and extracerebral blood flows and pressures in humans. Eur. J. Appl. Physiol..

[41] Ursino M, Lodi CA (1997). A simple mathematical model of the interaction between intracranial pressure and cerebral hemodynamics. J. Appl. Physiol..

[42] Ursino M, Ter Minassian A, Lodi CA, Beydon L (2000). Cerebral hemodynamics during arterial and CO(2) pressure changes: In vivo prediction by a mathematical model. Am. J. Physiol. Heart Circ. Physiol..

[43] Maas JJ (2015). Mean systemic filling pressure: Its measurement and meaning. Neth. J. Crit. Care.

[44] Majka M, Gadda G, Taibi A, Gałązka M, Zieliński P (2017). Protective properties of the arterial system against peripherally generated waves. Math. Biosci..

[45] Magnaes B (1976). Body position and cerebrospinal fluid pressure. Part 2: Clinical studies on orthostatic pressure and the hydrostatic indifferent point. J. Neurosurg..

[46] Kurazumi T, Ogawa Y, Morisaki H, Iwasaki K (2018). The effect of mild decrement in plasma volume simulating short-duration spaceflight on intracranial pressure. NPJ Microgravity..

[47] Nathoo N, Caris EC, Wiener JA, Mendel E (2011). History of the vertebral venous plexus and the significant contributions of Breschet and Batson. Neurosurgery.

[48] MathWorks Inc. (2019) Natick, MA. https://mathworks.com/products/simulink.html. Accessed 25 April 2020.

[49] Eklund A (2016). The pressure difference between eye and brain changes with posture. Ann. Neurol..

[50] Petersen LG, Petersen JC, Andresen M, Secher NH, Juhler M (2016). Postural influence on intracranial and cerebral perfusion pressure in ambulatory neurosurgical patients. Am. J. Physiol. Regul. Integr. Comp. Physiol..

[51] Mekis D, Kamenik M (2010). Influence of body position on hemodynamics in patients with ischemic heart disease undergoing cardiac surgery. Wien Klin. Wochenschr..

[52] Rangel-Castillo L, Gopinath S, Robertson CS (2008). Management of intracranial hypertension. Neurol. Clin..

[53] Lakin WD, Stevens SA, Penar PL (2007). Modeling intracranial pressures in microgravity: The influence of the blood-brain barrier. Aviat. Space Environ. Med..

[54] Foldager N (1996). Central venous pressure in humans during microgravity. J. Appl. Physiol..

[55] Grigoriev AI, Kotovskaya AR, Fomina GA (2011). The human cardiovascular system during space flight. Acta Astronaut..

[56] Valdueza JM, von Münster T, Hoffman O, Schreiber S, Einhäupl KM (2000). Postural dependency of the cerebral venous outflow. Lancet.

[57] Cirovic S, Walsh C, Fraser WD, Gulino A (2003). The effect of posture and positive pressure breathing on the hemodynamics of the internal jugular vein. Aviat. Space Environ. Med..

[58] Arbeille P, Provost R, Zuj K, Vincent N (2015). Measurements of jugular, portal, femoral, and calf vein cross-sectional area for the assessment of venous blood redistribution with long duration spaceflight (Vessel Imaging Experiment). Eur. J. Appl. Physiol..

