# Supplementary material for: Modelling physiology of haemodynamic adaptation in short-term microgravity exposure and orthostatic stress on Earth
Source: Sci Rep. 2021 Feb 25;11:4672. doi: 10.1038/s41598-021-84197-7 (PMC7907254; doi:10.1038/s41598-021-84197-7)
Supplement: Supplementary file 1 — Supplementary Information. [file 41598_2021_84197_MOESM1_ESM.docx]

**Modelling** **physiology of haemodynamic adaptation in short-term microgravity** **exposure and orthostatic stress on Earth**

**Parvin Mohammadyari^1^, Giacomo Gadda^2*^, and Angelo Taibi^1^**

1. University of Ferrara, Department of Physics and Earth Sciences, Ferrara, 44122, Italy
2. National Institute for Nuclear Physics (INFN), Section of Ferrara, Ferrara, 44122, Italy

*Corresponding author: Giacomo Gadda, PhD e-mail address: [gadda@fe.infn.it](mailto:gadda@fe.infn.it)

**SUPPLEMENTARY MATERIAL**

The model is an open loop mathematical modelling consists of two main parts: a 0-D and a 1-D algorithm ^1^. In the 0-D algorithm, the cerebrospinal autoregulation mechanisms and cerebral outflow were modelled according to the model of Ursino et al ^2,3^. The 1-D algorithm involves topology and propagation parameters (phase velocity and characteristic impedance) of the main vessels of the human arterial tree^4^.

***0-D venous algorithm***

In the 0-D networks, vasculature is considered as an analogous electric circuit, and the veins are electric elements with given values of capacitance and conductance ^5,6^. Moreover, the model takes into account the breathing effect on the drainage effect of the thoracic pump and central venous pressure ^2,7^. The model equations satisfy the momentum and mass conservation laws and describe the pressure-area relationship in accordance with literature and experimental data^1,2,8^.

Each vessel is considered as an electric element with a given value of resistance (R), capacitance (C), conductance (G) and Flow rate (Q) of the blood vessel according to the following equations ^2,5,7-9^.

$R=\frac{8\eta L}{\pi r^{4}}$ (1)

$G=\frac{1}{R}$ (2)

$C=\frac{increase in volume (\Delta V)}{increase in pressure (\Delta P)}$ (3)

$Q=\frac{1}{R}(P_{in}- P_{out})$ (4)

In the equations, the constant value ρ = 1060 kg/m^3^ , η = 3.8 × 10^-3^ Pa·s , L, r , *P_in_* and *P_out_* are the blood density, viscosity, length and radius of the vein, pressure inside the vessel and pressure outside the vessel, respectively.

*Venous Circuit*

To account for mass preservation in the whole 0-D model, the pressure change over time (dP/dt) is calculated according to the Kirchhoff law for each vessel of the whole network. The state equations used to build the jugular-vertebral circuit are the following:

${dP}_{vs}=\frac{1}{C_{vs}}[\left( P_{v}-P_{vs} \right)-\left( P_{vs}-P_{ic} \right)G_{o}-\left( P_{vs}-P_{jr3} \right)G_{jr3}-\left( P_{vs}-P_{jl3} \right)G_{jl3}-\left( P_{vs}-P_{c3} \right)G_{c3}-\left( P_{vs}-P_{vv} \right){(G}_{vv1}+G_{vv2})-\left( P_{vs}-P_{vp} \right)G_{vp}]$ (5)

${dP}_{vv}=\frac{1}{C_{vv}}[\left( P_{vs}-P_{vv} \right)(G_{vv1}+G_{vv2})-\left( P_{vv}-P_{azy} \right)G_{azy1}-\left( P_{vv}-P_{lv} \right)G_{lv}]$ (6)

${dP}_{jr2}=\frac{1}{C_{jr2}}[\left( P_{jr3}-P_{jr2} \right)G_{jr2}-\left( P_{jr2}-P_{c2} \right)G_{cjr2}-\left( P_{jr2}-P_{jr1} \right)G_{jr1}]$ (7)

${dP}_{jl2}=\frac{1}{C_{jl2}}[\left( P_{jl3}-P_{jl2} \right)G_{jl2}-\left( P_{jl2}-P_{c2} \right)G_{cjl2}-\left( P_{jl2}-P_{jl1} \right)G_{jl1}]$ (8)

${dP}_{jr1}=\frac{1}{C_{jr1}}[\left( P_{jr2}-P_{jr1} \right)G_{jr1}-\left( P_{jr1}-P_{svc1} \right)G_{svc1}]$ (9)

${dP}_{jl1}=\frac{1}{C_{jl1}}[\left( P_{jl2}-P_{jl1} \right)G_{jl1}-\left( P_{jl1}-P_{svc1} \right)G_{svc1}]$ (10)

${dP}_{vp}=\frac{1}{C_{vp}}[\left( P_{vs}-P_{vp} \right)G_{vp}-\left( P_{vp}-P_{azy} \right)G_{azy1}- \left( P_{vp}-P_{lv} \right)G_{lv}]$ (11)

${dP}_{dcl}=\frac{1}{C_{dc}}[\left( P_{ex}-P_{dcl} \right)G_{dc}-\left( P_{dcl}-P_{ejl} \right)G_{ej}]$ (12)

${dP}_{dcr}=\frac{1}{C_{dc}}[\left( P_{ex}-P_{dcr} \right)G_{dc}-\left( P_{dcr}-P_{ejr} \right)G_{ej}]$ (13)

${dP}_{jcl}=\frac{1}{C_{jc}}[\left( P_{ejl}-P_{jcl} \right)G_{jc}-\left( P_{jcl}-P_{jl3} \right)G_{jl3}]$ (14)

${dP}_{jcr}=\frac{1}{C_{jc}}[\left( P_{ejr}-P_{jcr} \right)G_{jc}-\left( P_{jcr}-P_{jr3} \right)G_{jr3}]$ (15)

${dP}_{ejl}=\frac{1}{C_{ej}}[\left( P_{ex}-P_{ejl} \right)G_{ej}-\left( P_{ejl}-P_{svc1} \right)G_{svc1}-\left( P_{ejl}-P_{jcl} \right)G_{jc}+\left( P_{ex}-P_{dcl} \right)G_{dc} ]$ (16)

${dP}_{ejr}=\frac{1}{C_{ej}}[\left( P_{ex}-P_{ejr} \right)G_{ej}-\left( P_{ejr}-P_{svc1} \right)G_{svc1}-\left( P_{ejr}-P_{jcr} \right)G_{jc}+\left( P_{ex}-P_{dcr} \right)G_{dc} ]$ (17)

${dP}_{azy}=\frac{1}{C_{azy}}[\left( P_{vv}-P_{azy} \right)G_{azy1}+\left( P_{lv}-P_{azy} \right)G_{lv}+\left( P_{vp}-P_{azy} \right)G_{vp}- \left( P_{azy}-P_{svc} \right)G_{azy2}]$ (18)

In this equation, *P*, *C* and *G* are the pressure, capacitance and conductance of the vessel in the upper and middle segment of the simulated veins in the Figure 1 (in the main text). The complete list of the variables is explained separately by Gadda et al.^1^.

Variable G has a switch-like function to simulate the collapsibility behaviour of the IJV. In the following the right side IJV segments G-function are stated, since the equations are the same for both right and left segments. Moreover, the thoracic pump effects on J1 and J2 are modelled by adding the thoracic pressure *P_thor_* in the G-function as represented in equations (20) and (21).

$G_{jr3}=k_{jr3}\left[ 1+\left( \frac{2}{\pi} \right)\arctan\left( \frac{P_{vs}- P_{j3ext}}{A_{J3}} \right) \right]^{2}$ (19)

$G_{jr2}=k_{jr2}\left[ 1+\left( \frac{2}{\pi} \right)\arctan\left( \frac{P_{jr3}- P_{j2ext}-(Pthor/2)}{A_{J2}} \right) \right]^{2}$ (20)

$G_{jr1}=k_{jr1}\left[ 1+\left( \frac{2}{\pi} \right)\arctan\left( \frac{P_{jr2}- P_{j1ext}- Pthor}{A_{J1}} \right) \right]^{2}$ (21)

***1-D Arterial algorithm***

The arterial network is designed by a compartmental method in which the vessels are modelled as monomode 1-D waveguides which simulate the pulse wave propagation through a network of electric transmission lines^32^. The parameters such as vessels length, mean blood velocity, topology of the arterial segments and the characteristic impedance of each segment are used to simulate the arterial hemodynamic characteristics ^4, 6^. The arterial 1-D compartments consist of 55 blocks that simulate the arterial tree and 11 blocks that simulate the Willis circle, respectively (76 arteries in total). Each block calculates the pressure pulse behaviour of a given artery by solving 1-D equations and considering the pressure as a transient wave pulse^4^. Arteries labelled with 48, 49, 54 and 55 are the posterior left tibial artery, anterior left tibial artery, posterior right tibial artery and anterior right tibial artery (see Figure 1).

In this model, ascending aorta is the first block fed by the ventricle pressure waveform that is generated by the heart. This pulse also models details about respiratory rate, hearth rate, systolic and diastolic blood pressure according to the reference pulse introduced by Guyton AC (1991)^10^. Each individual artery is designed to react to the input pulse wave accordingly to its characteristics. The simulated arteries and their modelled parameters are listed in Table 1. Such model considers the cardiac pulse propagation through the vessels. Vessel walls and blood are modelled by using Navier-Stokes equation, in which blood was considered as a non-compressive Newtonian fluid. The flow rate, blood pressure change, and mean lumen area can be calculated as a function of time (t) and space (x) by computing the volume and change of volume for each compartment through the equations below ^4,8,11,12^:

$Q(x,t)=A\times\bar{V}(x,t)$ (22)

$\frac{\partial Q}{\partial t}+\frac{\partial}{\partial x}(\frac{Q^{2}}{A})+\frac{A}{\rho}\frac{\partial P}{\partial x}=\frac{f}{\rho}$ (23)

$\frac{\partial A}{\partial t}+\frac{\partial Q}{\partial z}=0$ (24)

$P=P_{0}+\frac{E\pi R_{0}h}{A}(\sqrt{\frac{A}{A_{0}}}-1)+\frac{k\pi R_{0}h}{A}\frac{1}{2\sqrt{AA_{0}}}\frac{\partial A}{\partial t}$ (25)

where A is the internal area of the vessel at a given section, and *f* (*x*, *t*) is the frictional force per unit length. Parameters E and k are the effective Young modulus and elastic component of the vessel wall (subscript 0 refers to reference values). These equations satisfy the momentum and mass conservation laws and describe the pressure-area relationship.

At any artery bifurcation, a parent artery (a) and two branched daughter arteries (d1, d2) are defined. The distribution of incoming fluid between daughters depends on the reflection and transmission coefficients, that can be calculated from the mechanical characteristics of the vessels such as impedance (Z), as described by equations 26 and 27 ^4,8^.

$Z=\frac{\rho c}{A_{0}}$ (26)

$R_{f}^{a}=\frac{\left( Z^{a} \right)^{-1}-\left( Z^{d1} \right)^{-1}-\left( Z^{d2} \right)^{-1}}{\left( Z^{a} \right)^{-1}+\left( Z^{d1} \right)^{-1}+\left( Z^{d2} \right)^{-1}}$ (27)

where c is the mean velocity of blood at the parent artery.

| **No.** | **Vessel** | **L** | **C** | **Z_0_** | **t_d_** | **R_f_** |
| --- | --- | --- | --- | --- | --- | --- |
| 56 | Basilar | 0.0290 | 15.8377 | 2.0170e+09 | 0.0018 | - |
| 57 | Posterior cerebral segment A (R) | 0.0050 | 6.0107 | 4.6739e+09 | 0.0003 | - |
| 58 | Posterior cerebral segment A (L) | 0.0050 | 6.0107 | 4.6739e+09 | 0.0003 | - |
| 59 | Posterior comm (R) | 0.0150 | 19.1237 | 2.5566e+10 | 0.0008 | - |
| 60 | Posterior comm (L) | 0.0150 | 19.1237 | 2.5566e+10 | 0.0008 | - |
| 61 | Anterior common A | 0.003 | 19.6477 | 2.6267e+10 | 0.0002 | - |
| 62 | Anterior cerebral segment A (R) | 0.0120 | 15.8681 | 3.8743e+09 | 0.0008 | - |
| 63 | Anterior cerebral segment A (L) | 0.0120 | 15.8681 | 3.8743e+09 | 0.0008 | - |
| 64 | External carotid (R) | 0.118 | 11.9252 | 1.7714e+09 | 0.0099 | - |
| 65 | External carotid (L) | 0.118 | 11.9252 | 1.7714e+09 | 0.0099 | - |
| 66 | Posterior cerebral segment B (R) | 0.0860 | 5.8603 | 4.8081e+09 | 0.0054 | 0.6548 |
| 67 | Posterior cerebral segment B (L) | 0.0860 | 5.8603 | 4.8081e+09 | 0.0054 | 0.6548 |
| 68 | Ophthalmic artery (R) | 0.03 | 17.0360 | 1.1620e+10 | 0.0018 | 0.8188 |
| 69 | Ophthalmic artery (L) | 0.03 | 17.0360 | 1.1620e+10 | 0.0018 | 0.8188 |
| 70 | Middle cerebral artery (R) | 0.03 | 18.4010 | 1.7084e+10 | 0.0016 | 0.8535 |
| 71 | Middle cerebral artery (L) | 0.03 | 18.4010 | 1.7084e+10 | 0.0016 | 0.8535 |
| 72 | Cerebral artery (R) | 0.0580 | 18.1695 | 9.4886e+09 | 0.0032 | 0.7563 |
| 73 | Cerebral artery (L) | 0.0580 | 18.1695 | 9.4886e+09 | 0.0032 | 0.7563 |
| 74 | Internal carotid (R) | 0.0590 | 11.2683 | 1.1624e+09 | 0.0052 | - |
| 75 | Internal carotid (L) | 0.1480 | 11.2683 | 1.1624e+09 | 0.0052 | - |
| 76 | Facial A | 0.04 | 17.4567 | 5.8345e+09 | 0.0023 | 0.6552 |

**Table 1.** List of arteries and parameters modelled in the brain including the Willis circle tree (see Fig. 1 middle green box). R: right artery; L: left artery. No. is the vessel number in the model, L is the length of the vessel, c is the mean velocity, Z_0_ is the characteristic impedance, t_d_ is the time delay, and R_f_ is the reflection coefficient of the peripherals. Arteries from 1 to 55 are already listed in Majka et al^4^.

**Reference list**

1. Gadda, G. *et al.* A new hemodynamic model for the study of cerebral venous outflow. *Am. J. Physiol. Heart Circ. Physiol.* **308,** H217–H231, DOI:<https://doi.org/10.1152/ajpheart.00469.2014> (2015).
2. Ursino, M. & Lodi, C. A. A simple mathematical model of the interaction between intracranial pressure and cerebral hemodynamics. *J. Appl. Physiol.* **82,** 1256–1269, DOI:<https://doi.org/10.1152/jappl.1997.82.4.1256> (1997).
3. Ursino, M., Ter Minassian, A., Lodi, C. A. & Beydon, L. Cerebral hemodynamics during arterial and CO(2) pressure changes: in vivo prediction by a mathematical model. *Am. J. Physiol. Heart Circ. Physiol.* **279,** H2439–H2455, DOI:https://doi.org/10.1152/ajpheart.2000.279.5.H2439 (2000).
4. Majka, M., Gadda, G., Taibi, A., Gałązka, M. & Zieliński, P. Protective properties of the arterial system against peripherally generated waves. *Math. Biosci.* **286,** 16–21, DOI:<https://doi.org/10.1016/j.mbs.2017.01.007> (2017).
5. Gisolf, J. *et al.* Human cerebral venous outflow pathway depends on posture and central venous pressure. *J. Physiol.* **560,** 317–327, DOI:<https://doi.org/10.1113/jphysiol.2004.070409> (2004).
6. Diaz-Artiles, A., Heldt, T. & Young, L.R. Effects of artificial gravity on the cardiovascular system: computational approach. *Acta Astronaut.* **126,** 395–410, DOI:https://doi.org/10.1016/j.actaastro.2016.05.005 (2016).
7. Gadda, G., Majka, M., Zieli[ń](https://www.ncbi.nlm.nih.gov/pubmed/?term=Zieli%C5%84ski%20P%5BAuthor%5D&cauthor=true&cauthor_uid=30171350)ski, P., Gambaccini, M. & Taibi, A. A multiscale model for the simulation of cerebral and extracerebral blood flows and pressures in humans. *Eur. J. Appl. Physiol.* **118,** 2443-2454, DOI:<https://doi.org/10.1007/s00421-018-3971-3> (2018).
8. Zhang, X., Noda, S., Himeno, R. & Liu H. Gravitational effects on global hemodynamics in different postures: a closed-loop multiscale mathematical analysis. *Acta Mech. Sinica,* **33,** 595–618, DOI:https://doi.org/10.1007/s10409-016-0621-z (2017).
9. Olufsen, M., Tran, H. & Ottesen, J. Modeling cerebral blood flow control during posture change from sitting to standing. *Cardiovasc. Eng. Techn.* **4,** 47–58, DOI:https://doi.org/10.1023/B:CARE.0000025122.46013.1a (2004).
10. Guyton A. C. *The veins and their functions*. In: Textbook of medical physiology. Saunders, Philadelphia (1991).
11. Qureshi, M.U. *et al.* Numerical simulation of blood flow and pressure drop in the pulmonary arterial and venous circulation. *Biomech. Model. Mechanobiol.* **13,** 1137–1154, DOI:https://doi.org/10.1007/s10237-014-0563-y (2014).
12. Larrabide, I. *et al.* HeMoLab – Hemodynamics Modelling Laboratory: an application for modelling the human cardiovascular system. *Comput. Biol. Med.* **42,** 993–1004, DOI:https://doi.org.10.1016/j.compbiomed.2012.07.011 (2012).
